# ALB3 Insertase Mediates Cytochrome *b*_6_ Co-translational Import into the Thylakoid Membrane

**DOI:** 10.1038/srep34557

**Published:** 2016-10-04

**Authors:** Jarosław Króliczewski, Małgorzata Piskozub, Rafał Bartoszewski, Bożena Króliczewska

**Affiliations:** 1 Laboratory of Chemical Biology, Faculty of Biotechnology, University of Wrocław, Wrocław Poland; 2 Amplicon Sp. z o. o., Wrocław, Poland; 3 Faculty of Biotechnology, University of Wrocław, Wrocław, Poland; 4 Department of Biology and Pharmaceutical Botany, Medical University of Gdansk, Gdansk, Poland; 5 Department of Animal Physiology and Biostructure, Faculty of Veterinary Medicine, Wrocław University of Environmental and Life Sciences, Wrocław, Poland

## Abstract

The cytochrome *b*_6_* f* complex occupies an electrochemically central position in the electron-transport chain bridging the photosynthetic reaction center of PS I and PS II. In plants, the subunits of these thylakoid membrane protein complexes are both chloroplast and nuclear encoded. How the chloroplast-encoded subunits of multi-spanning cytochrome *b*_6_ are targeted and inserted into the thylakoid membrane is not fully understood. Experimental approaches to evaluate the cytochrome *b*_6_ import mechanism *in vivo* have been limited to bacterial membranes and were not a part of the chloroplast environment. To evaluate the mechanism governing cytochrome *b*_6_ integration *in vivo,* we performed a comparative analysis of both native and synthetic cytochrome *b*_6_ insertion into purified thylakoids. Using biophysical and biochemical methods, we show that cytochrome *b*_6_ insertion into the thylakoid membrane is a non-spontaneous co-translational process that involves ALB3 insertase. Furthermore, we provided evidence that CSP41 (chloroplast stem–loop-binding protein of 41 kDa) interacts with RNC-cytochrome *b*_6_ complexes, and may be involved in cytochrome *b*_6_ *(petB)* transcript stabilization or processing.

Photosynthetic electron transport is accomplished by three hetero-oligomeric integral oxygenic photosynthetic membrane protein complexes: Photosystems I (PS I) and II (PS II), and the cytochrome *b*_6_ *f* complex. The 220-kDa cytochrome *b*_6_* f* complex dimer occupies an electrochemically central position in the electron-transport chain^1^. Hence, cytochrome *b*_6_ *f* provides an electronic connection between the two photosynthetic reaction centers of PS I and PS II, allowing linear electron transfer from the H_2_O electron donor to the NADP (nicotinamide-adenine dinucleotide phosphate) acceptor^2^. Furthermore, cytochrome *b*_6_ *f* and PS I complex participate in cyclic electron transfer, which generates an electrochemical proton gradient across the thylakoid membrane without net production of reducing equivalents^3^. In plants, these electron-transfer chain protein complexes are located in chloroplast thylakoid membranes, while their subunits are encoded by both nuclear and chloroplast genomes^4^. The proper thylakoid membrane assembly of PS I, PS II and cytochrome *b*_6_ *f* requires numerous regulatory factors for coordinated transport, insertion and assembly of these complexes subunits from both chloroplast and nuclear origin^5^. Although the electron-transfer chain function and structure have been extensively studied, the mechanism governing the assembly of these complexes in the thylakoid membrane is less understood. Specifically, little is known how their chloroplast-encoded subunits are targeted and inserted into the thylakoid membrane.

However, for the import into the thylakoid membrane of proteins from both nuclear and chloroplast origin, four independent precursor-specific transport pathways had been proposed (classified on the basis of their energy and stromal factor requirements)^6^. These four pathways have been categorized as “spontaneous”, signal recognition particle (SRP), secretory (Sec), and twin-arginine translocase-dependent (ΔpH/Tat)^7^. Integration of proteins into thylakoid membranes relies not only on the membrane translocation machinery, but also on the chloroplast stromal fraction. The Sec pathway requires the translocation ATPase and SecA proteins^8^. The cpSRP pathway uses GTP, cpSRP54 and cpSRP43 to target proteins to the thylakoid membrane, but the Tat pathway uses a *trans*-thylakoid pH gradient as its sole energy source^7^. All of these are found in the soluble stromal fraction. However, the “spontaneous” pathway does not seem to require any soluble factors or energy source for protein insertion into membrane^9^. The majority of proteins incorporated into the thylakoid membrane utilize the spontaneous or SRP pathway, while protein transport through the thylakoid membrane is mediated by the Sec or ΔpH/Tat pathway^6^.

Commonly, nuclear-encoded multi-spanning proteins are targeted to the thylakoid membrane by hydrophobic amino acid sequences from either their transmembrane segments or from a cleavable signal sequence (ss)^10^. Whereas, the spontaneous pathway seems to be the mainly utilized for the import of single-span proteins^7^. Importantly, the spontaneous mechanism was also shown to be active for some of nuclear-encoded multi-spanning thylakoid membrane proteins including the PsaK and PsaG subunits of PS I^11^,^12^.

The chloroplast-encoded cytochrome *b*_6_ binds a one covalently c-type haem as well as two non-covalently b-type haems and consists of four transmembrane helices, while the signal for this integral protein integration with thylakoid membrane remains unknown^13^,^14^. In the current model for assembly of cytochrome *b*_6_ *f* complex, the first step involves the transcriptional activation of the chloroplast *petBD* operon (encoding cytochrome *b*_6_ and subunit IV)^13^. Following the transcription, the *petB* and *petD* mRNAs are translated into the polypeptides that undergo insertion into the membrane and form the polytopic monomeric core of the cytochrome *b*_6_ *f* complex. In the next step the monomers form a dimer (CS) which is stabilized by lipids, and simultaneously a Rieske ISP-cytochrome *f* sub-complex (RF) is formed. This sub-complex then interacts with the CS to form a cytochrome *b*_6_-subunit IV-ISP-cytochrome *f* sub-complex (CSRF). Regardless of the formation of the CSRF complex, small subunits (Pet G, L, M and N) form an additional sub-complex which may interact with the RF^15^. At last fully functional cytochrome *b*_6_* f* complex is formed.

Hence, cytochrome *b*_6_ *f* complex assembly process requires a complex coordination between transcription, translation, chloroplast membrane transport, membrane insertion and sub-complexes assembly.

To date, experimental approaches to evaluate the cytochrome *b*_6_ import mechanism *in vivo* were limited to bacterial membrane and therefore did not involve the chloroplast environment^16^,^17^,^18^,^19^,^20^.

Hence, the objective of the present study was to examine the mechanism governing cytochrome *b*_6_ integration into the thylakoid membrane. Our comparative analysis of both native and synthetic cytochrome *b*_6_ revealed that an unfolded cytochrome *b*_6_ can be anchored into the thylakoid membrane by hydrophobic interactions that can be removed by chaotropic action. Hence, we excluded the spontaneous pathway for insertion of cytochrome *b*_6_ into the thylakoid membrane *in vivo*. Furthermore, our results indicate that the proper integration of cytochrome *b*_6_ is co-translationally mediated by other proteins. Indeed, we determined ALB3 insertase is a crucial protein for cytochrome *b*_6_ insertion into thylakoid membrane. Furthermore, we identified cpFtsY and CPS41 (chloroplast stem–loop-binding protein of 41 kDa) as other proteins involved in this process. These data are not limited to cytochrome *b*_6_, but also provide a new insight into the mechanisms involved in the insertion of integral membrane proteins integration into the thylakoid membrane.

## Results

The primary aim of this work was to analyse the mechanism by which cytochrome *b*_6_ inserts into the thylakoid membrane. For these experiments, we used import assays for the insertion of different variants of cytochrome *b*_6_ proteins into purified Pea thylakoids. Our main criterion for correct insertion was that the cytochrome *b*_6_ has to be integrated with the membrane and thus cannot be extracted along with extrinsic, non-inserted proteins^21^. Furthermore, the cytochrome *b*_6_ has to be properly oriented in the membrane, with the C- terminus and N-terminus at the stromal side of the thylakoid membrane.

### Cytochrome *b*_6_ integration with thylakoid membrane is not a spontaneous process

**Figure 1:**
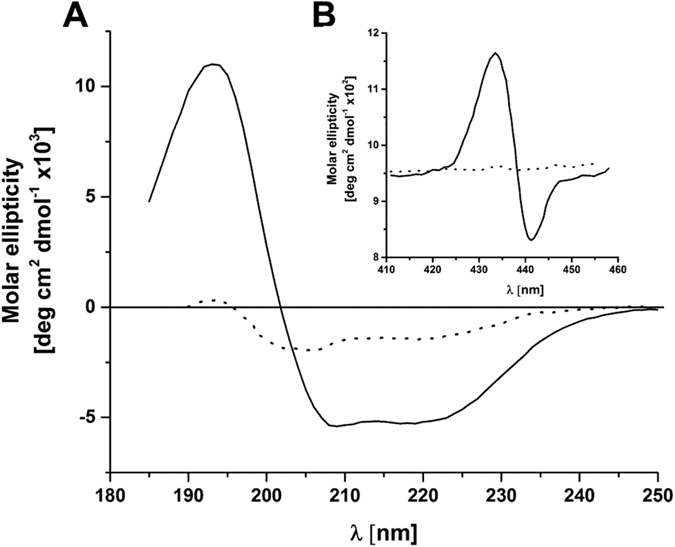
Circular dichroism spectroscopy of native cytochrome *b*_6_. (**A**) Native cytochrome *b*_6_ (solid line), denatured native cytochrome (dot line), Spectra are shown for the protein in buffer containing DDM. (**B**) The description is the same as in panel A.

To follow the native cytochrome *b*_6_ integration with the Pea thylakoid membrane, the protein was purified from *Synechocystis sp.* PCC 6803 as described in ref. 22 and solubilised in the presence of n-dodecyl-β-D-maltoside (DDM). As shown in Supplemental Fig. 1, an amino acid consensus between cytochrome *b*_6_ protein sequences from Pea and Synechocystis sp. PCC 6803 was above 87% and positions with identical amino acid residues were above 79%. The circular dichroism (CD) spectra of isolated native cytochrome *b*_6_ indicated a high proportion of the α-helical structure characterised by negative maxima at 208 and 222 nm (Fig. 1A and Table 1). Following the import assay of native cytochrome *b*_6_, the protein integration with thylakoid membrane was tested with use of urea as chaotropic agent. Urea has been proven to be effective in removing extrinsic, non-inserted proteins from the thylakoid membranes^21^. Chaotropic agents are co-solutes that disrupt the van der Waals forces and hydrogen-bonding network between water molecules and reduce the stability of the native state of proteins by weakening the hydrophobic effect.

**Figure 2:**
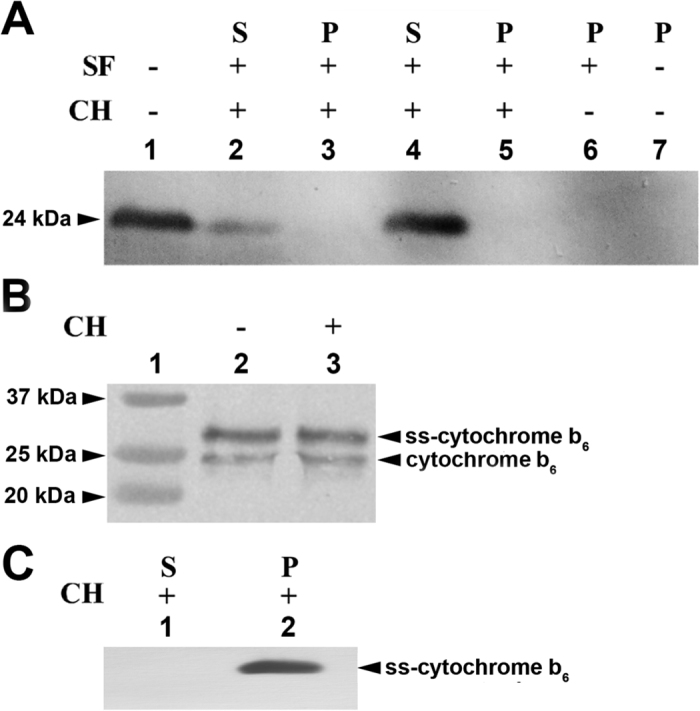
*In vitro* import of cytochrome *b*_6_ into thylakoid membrane. (**A**) The integration of the cytochrome *b*_6_ into the thylakoid membrane in the presence or absence of stromal fraction (SF) was analysed with Western blot. Urea was used as chaotropic agents (CH). Lane 1, purified native cytochrome *b*_6_ as a control; lanes 2 and 3, supernatant (S) and membrane pellet (P) after insertion of native cytochrome *b*_6_; lanes 4–7, supernatant and membrane pellet after insertion of denatured cytochrome *b*_6_. Cytochrome *b*_6_ was isolated from *Synechocystis sp.* PCC 6803, biotin labelled and anti-biotin antibodies was used for detection. (**B**) Lane 1, molecular weight standard; Lanes 2–3, membrane fraction after ss-cytochrome *b*_6_ insertion with and without chaotropic extraction, respectively. Antibodies against N-terminal residues of cytochrome *b*_6_ were used (10 μg of total protein per each lane was applied). (**C**) Lanes 1 and 2, supernatant (S) and membrane pellet (P) after insertion of ss-cytochrome *b*_6_. Urea was used as a chaotropic agents (CH) and anti-biotin antibodies were used for protein detection. All the experiments were repeated twice and 10 μg of total protein per each lane was applied.

In order to distinguish the inserted protein from the original cytochrome *b*_6_ located in the isolated thylakoid membrane, prior to insertion assays, the exogenous cytochrome *b*_6_ was biotin labelled. Notably, thylakoid-associated native cytochrome *b*_6_ (isolated from *Synechocystis sp*. PCC 6803) was almost entirely chaotropic-extractable by a mild concentration of urea (4 M) and not detectable in the membrane fraction (Fig. 2A lane 3).

Furthermore, in a control experiment, after insertion of cytochrome *b*_6_, we followed the native cytochrome folding during chaotropic extraction (Supplemental Fig. S2), and this protein was resistant to the treatment as reported^23^. As shown in Fig. 2B,C, both of the imported proteins, ss-cytochrome *b*_6_ and the native cytochrome *b*_6_, showed resistance to 4 M urea extraction. Hence, the native cytochrome *b*_6_ could not incorporate with thylakoid membranes spontaneously nor posttranslationaly through stromal proteins of Sec pathway.

A chemical denaturation of isolated cytochrome *b*_6_ was followed by UV-Vis spectroscopy and a circular dichroism analysis at 222 nm. The secondary structure of native cytochrome *b*_6_ was lost upon unfolding in the presence of GuHCl (guanidine hydrochloride). The GuHCl treatment led to a relatively flat spectrum indicating a substantial loss of secondary structure (Fig. 1A and Table 1). Furthermore, in the visible CD spectrum of the denatured cytochrome *b*_6,_ a loss of heme and no Cotton effects in the Soret-band region were observed (Fig. 1B), as reported previously^24^.

Similarly, following the import assay of unfolded cytochrome *b*_6_, the protein integration with thylakoid membranes was tested with use of a chaotropic agent. As shown on Fig. 2A (lanes 4–7), independent of stromal fraction presence, no unfolded cytochrome *b*_6_ band was observed in thylakoid membranes after urea treatment. Hence, the denaturized cytochrome *b*_6_ neither integrated with thylakoid membranes utilizing spontaneous nor posttranslational Sec pathways. However under denaturing conditions, noncovalently bound haems dissociate from proteins^25^, although this did not affect the shape of the electrophoretic band for cytochrome *b*_6_ (Fig. 2A lane 4).

**Figure 3:**
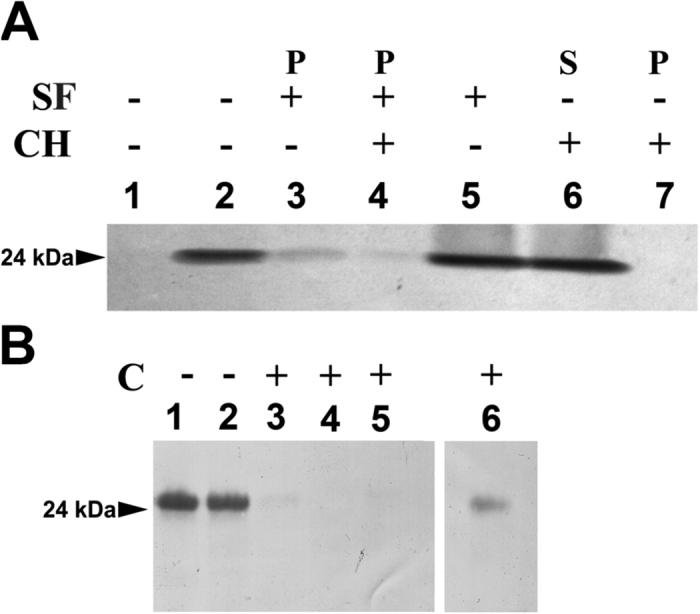
Thylakoid membrane fractions after insertion of spinach apocytochrome *b*_6_ and treatment with chaotropic agents (CH). (**A**) The integration of the cytochrome *b*_6_ into the thylakoid membrane in the presence or absence of stromal fraction (SF) was analysed with Western blot. Lane 1, thylakoid membrane; lane 2, purified apocytochrome *b*_6_; lane 3 pelleted membrane fraction with inserted denatured (unfolded) protein; lane 4, pelleted membrane fraction with inserted denatured protein after chaotropic treatment with urea; lane 5, control, membrane fraction with inserted into membrane ss-apocytochrome *b*_6_; lanes 6 and 7, supernatant and membrane pellet, respectively after centrifugation of refolded apocytochrome *b*_6_ and inserted into membrane. The experiments were repeated twice and 10 μg of total protein per lane was applied. (**B**) Thylakoid membrane fractions after insertion of spinach ss-apocytochrome *b*_6_ and treatment with carboxypeptidase B. Lane 1, purified ss-apocytochrome *b*_6_; lane 2, thylakoid membrane with inserted ss-apocytochrome *b*_6_; lane 3, membrane treated with carboxypeptidase B (depicted with (C) after protein insertion; lanes 4 and 5, membrane and supernatant fraction with inserted denatured protein after carboxypeptidase B treatment; lane 6, supernatant fraction similar to lane 5, but membrane with inserted denatured protein was treated with urea and carboxypeptidase B, and an antibody against N-terminus of cytochrome *b*_6_ was used. Cytochrome *b*_6_ was biotin labelled and anti-biotin antibodies were used for detection with the exception of (**B**) line 6.

Spectroscopic analysis was conducted for integration into the thylakoid membranes of *E. coli* expressed spinach apocytochrome *b*_6_^18^. Refolding of apocytochrome *b*_6_ was monitored by far-UV CD spectroscopy at 222 nm (Supplemental Fig. S3). As shown on Fig. 3A, in contrast to denaturized *Synechocystis sp*. PCC 6803 cytochrome *b*_6_, the denaturized apocytochrome *b*_6_ was only partially imported to the thylakoids (lane 3). Furthermore, both denaturized and refolded spinach apocytochrome *b*_6_ was sensitive to 4 M urea extraction (Fig. 3 lanes 4, 6 and 7) as well as by others chaotropes (Supplemental Fig. S4A). Hence spinach apocytochrome *b*_6_ did not integrate into the thylakoid membranes spontaneously, as well as the cyanobacteria native protein. On the other hand, ss-apocytochrome *b*_6_ was imported into the thylakoid membrane and properly oriented in the membrane with C-terminus and N-terminus at stromal side of thylakoid membrane (Fig. 3B lanes 2 and 3) and antibodies against cpSecY prevent cpSecA-dependent protein translocation into membrane by the Sec pathway^26^,^27^,^28^ (Supplemental Fig. S5).

In the case of denaturized cytochrome *b*_6_, no biotin signal was detected after carboxypeptidase B treatment in both the membrane pellet and supernatant (Fig. 3B lanes 4 and 5), although the N-terminal signal of denaturized cytochrome *b*_6_ was observed in the supernatant.

### PsbW integrates spontaneously with the isolated thylakoid membranes

In order to validate our cytochrome *b*_6_ insertion assays, we followed the thylakoid membrane integration of the cytosolic single span subunit W of PS II (PsbW), since this protein inserts into the thylakoid membrane by an apparently spontaneous pathway^29^. PsbW was used as an independent control for our experimental model. Since our experiments test whether the insertion of cytochrome *b*_6_ into the thylakoid membrane occurs spontaneously, the spontaneous insertion of mature PsbW into the thylakoid membrane observed in the same experimental model validates our methodological approach.

**Figure 4:**
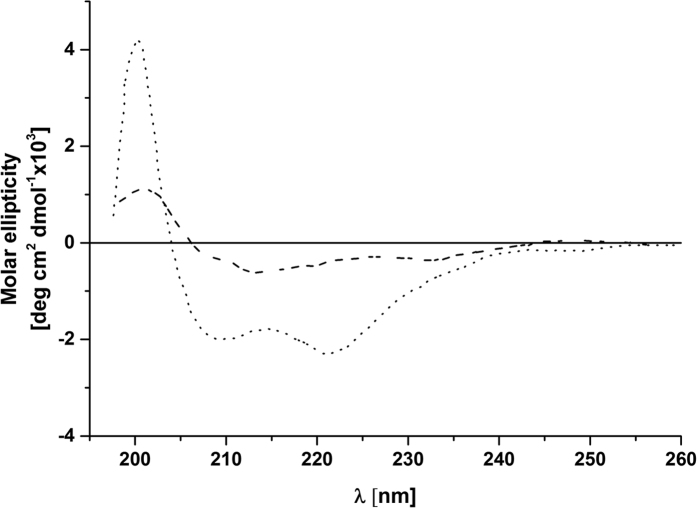
Circular dichroism spectroscopy of PsbW in aqueous buffer (unfolded) and DDM micelles (refolded). Spectra are shown for the protein in aqueous buffer (dashed line) and after incorporation into DDM micelles (dotted line).

In order to probe the structure of the soluble form of the synthetic PsbW protein, biophysical analyses by CD and MS (mass spectroscopy) were performed. The CD spectra of denatured and DDM refolded PsbW differed significantly in secondary structure, indicating that the PsbW protein forms a transmembrane α-helix in hydrophobic environments of DDM (Fig. 4). The obtained spectra were overall in good agreement with previous studies^29^. The determined masses of the denatured and refolded protein protein’s (base on mass spectra), allowed us to establish the oligomeric stoichiometry of PsbW complex before insertion into the membrane. The MS analysis observed for biotynylated PsbW (6394.65 Da) agreed with the theoretical molecular weight of the monomeric species (6055.49 Da). Furthermore, MS and CD analysis showed that these proteins exist mainly as monomers (~89% for monomers and ~11% for oligomers).

**Figure 5:**
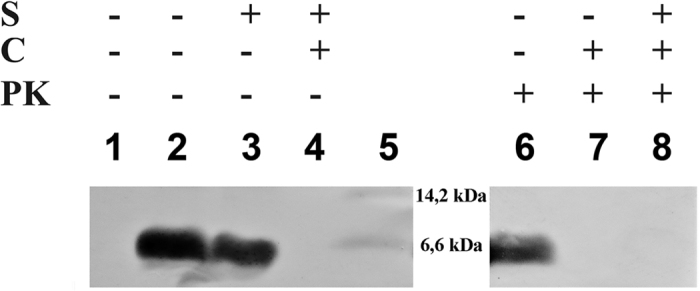
Thylakoid membrane fractions after insertion of PsbW. The integration of the PsbW into the thylakoid membrane the presence or absence of stromal fraction was analysed by Western blot. Lane 1, thylakoid membrane before insertion; lanes 2–4 and 6–8, thylakoid membrane after insertion of PsbW; and lane 5, molecular weight standard. Antibodies against biotin were used for immunodetection. C - membrane treated with carboxypeptidase B after protein insertion, PK - membrane treated before protein insertion with proteinase K. On each lane, 10 μg of protein was applied. Identification of psbW protein in Western blot was also confirmed using MS.

As shown on Fig. 5, our *in vitro* experiments verified that synthetic PsbW is indeed spontaneously inserted into the isolated thylakoid membrane. The thylakoid import assays showed that the PsbW inserted into the thylakoid membranes and sorted efficiently also in an absence of a stromal fraction (quantified by densitometry analysis) and in the presence of apyrase (Supplemental Fig. S6). Apyrase is an ATP-diphosphohydrolase that catalyses the sequential hydrolysis of ATP to ADP and ADP to AMP and releases inorganic phosphate and prevents *SecA* de-insertion and further translocation across the thylakoid membrane by the ATP-dependent Sec pathway.

Following the incubation of DDM vesicles of PsbW with carboxypeptidase B that catalyzes the hydrolysis of the basic amino acids from the C-terminal position of polypeptides (Fig. 5, lanes 4 and 7), the biotin labelled C-terminus of PsbW was completely sensitive to digestion and no biotin signal was detected after carboxypeptidase B treatment of PsbW. Hence incorporation of PsbW into the membrane was direct, with the N-terminus and the C-terminus on the opposite sides of the membrane. Furthermore, the thylakoid membranes proteinase K pretreatment did not inhibit insertion of the PsbW protein (Fig. 5, lane 6). Finally, the membrane integrated PsbW was completely insensitive to removal (Supplemental Fig. S7). These results confirmed previous reports of spontaneous insertion of PsbW into the thylakoid membrane^29^,^30^,^31^.

### SRP-related chloroplast proteins are responsible for the cytochrome *b*_6_ integration into thylakoid membrane

The results of the import assays questioned both spontaneous and Sec-dependent mechanisms for cytochrome *b*_6_ import into thylakoid membrane. Furthermore, since efficient import was observed for unfolded cytochrome *b*_6_ only, the involvement of posttranslational SRP mechanism seemed unlikely. Hence, to confirm directly that the cytochrome *b*_6_ is a chloroplast protein targeted in a GTP-dependent (guanosine triphosphates) process termed co-translational translocation, chloroplast import experiments were performed using cell free *in vitro* system.

**Figure 6:**
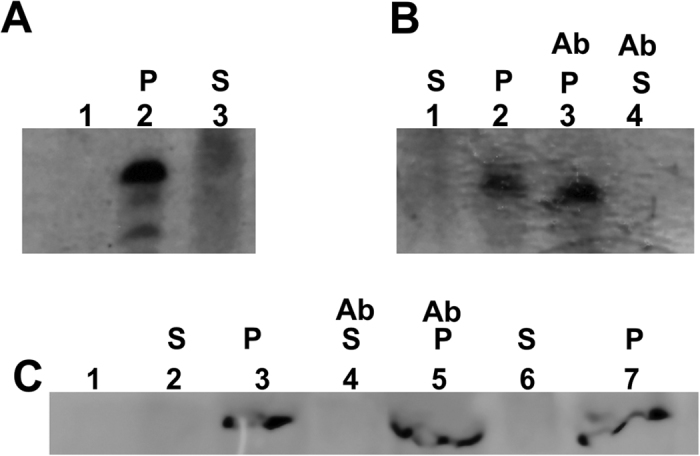
Autoradiograph of cytochrome *b*_6_ expressed in cell-free assay in the presence of thylakoid membrane and stroma. (**A**) Lane 1, thylakoid membrane as a control; lanes 2 and 3, translation of cytochrome *b*_6_ in the presence of thylakoid membrane and stromal fraction, supernatant (S) and membrane pellet (P) after fractionation; (**B**) Lanes 1 and 2 translation of cytochrome *b*_6_ in the presence of thylakoid membrane and stromal fraction, supernatant (S) and membrane pellet (P) after fractionation; lane 3 and 4, translation of cytochrome *b*_6_ in the presence of thylakoid membrane, stroma and cpSecY antibody, membrane pellet (P) and supernatant after fractionation; (**C**) Lane 1, thylakoid membrane as a control; lane 2 and 3, translation of cytochrome *b*_6_ in the presence of thylakoid membrane and stromal fraction, supernatant (S) and membrane pellet (P) after fractionation, lane 4 and 5, translation of cytochrome *b*_6_ in the presence of thylakoid membrane, stroma and cpSecY antibody, membrane pellet (P) and supernatant (S) after fractionation; lane 6 and 7 same as in lane 2 and 3 but endogenous RNA in stroma was removed by enzymatic digestion before use in translation reaction (reaction were performed in the presence of RNasin ribonuclease inhibitor).

Cell-free native spinach cytochrome *b*_6_ expression was performed in the linked system and transcription and translation reactions were separated. Translations were carried out in the presence of thylakoid membrane or thylakoid membrane with a stroma fraction. As shown in Fig. 6 (lanes 2 and 3), the translation product was detected in the thylakoid membrane. Pretreatment of thylakoids and stroma with proteinase K (Fig. 6, lane 4) prevented insertion of cytochrome *b*_6_ into membrane due to degradation of thylakoid and stromal translocation proteins. Furthermore, in Fig. 6, lane 5, a significant level of insertion was achieved in the presence of cpSecY antibody. Antibodies against cpSecY prevent cpSecA-dependent protein translocation into membrane by the Sec pathway^26^,^27^,^28^, suggesting that integration of the cytochrome *b*_6_ is Sec-independent. Following the import assay of native cytochrome *b*_6_, protein integration with thylakoid membrane was tested with the use of a chaotropic agent (Supplemental Fig. S4B). Furthermore, the membrane integrated cytochrome *b*_6_ was completely insensitive to removal by urea, KSCN and NaOH.

Next, to identify chloroplast proteins that could govern cytochrome *b*_6_ import a chemical cross-linking analysis combined with a mass spectrometry approach was applied. Cytochrome *b*_6_ cell-free translations were carried out in the presence of thylakoid membrane with a stroma fraction. Interacting proteins were identified with the cytochrome *b*_6_ targeted ribosome-nascent chain complexes (RNCs) after immunoprecipitation of cross-linked proteins with an antibody against cytochrome *b*_6_. These proteins were identified with MS using Mascot Distiller software as shown in Table 2 and Supplemental Table S1. A MS analysis of proteins co-immunoprecipitated with an antibody against cytochrome *b*_6_ without crosslinker was used as a control of the specificity of the cross-linking (Supplemental Table S2).

The identified proteins were previously recognized either as crucial for SRP-mediated co-translational transport into thylakoid membrane (cpSRP54, cpFtsY, and ALB3) or for untranslated plastid mRNA stabilization (CSP41). The cytochrome *b*_6_ elongating nascent chain is known to interact with the cpSRP54 but not with cpSecY^19^. Our results indicate that targeting and insertion of cytochrome *b*_6_ protein into the thylakoid membrane occurs through co-translational SRP pathway *in vivo*.

**Figure 7:**
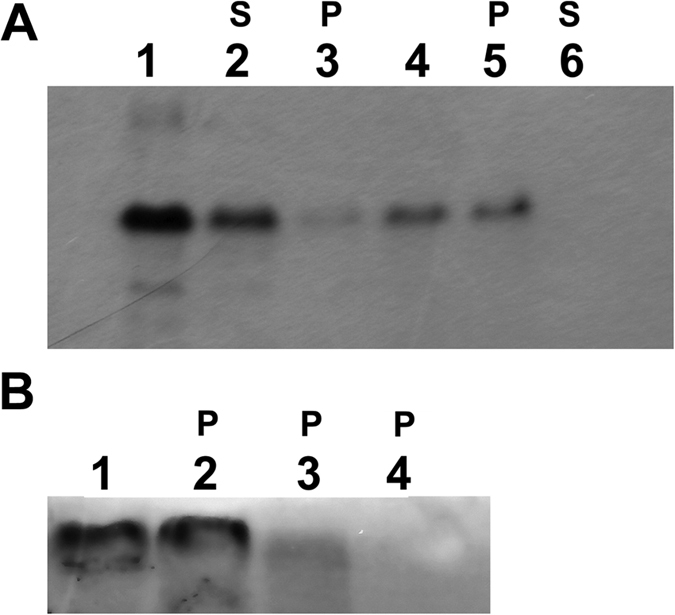
Autoradiograph of cytochrome *b*_6_ expressed in cell free assay in the presence of thylakoid membrane and stroma. (**A**) Lane 1, translation of cytochrome *b*_6_ - control; lane 2 and 3, translation of cytochrome *b*_6_ in the presence of thylakoid membrane, stromal fraction and ALB3 antibody, supernatant (S) and membrane pellet (P) after fractionation; lane 4, translation of cytochrome *b*_6_ in the presence of thylakoids membrane and stroma; lane 5 and 6, translation of cytochrome *b*_6_ in the presence of thylakoid membrane and stromal fraction, supernatant (S) and membrane pellet (P) after fractionation. (**B**) Lane 1, translation of cytochrome *b*_6_, membrane pellet fraction after fractionation - control; lane 2, translation of cytochrome *b*_6_ in the presence of 0.5 mM non-hydrolysable ATP analogue (AMP-PNP, 5′ adenylylimidodiphosphate), membrane pellet; lane 3, translation of cytochrome *b*_6_ in the presence of 0.1 mM GMP-PNP, membrane pellet; lane 4, translation of cytochrome *b*_6_ in the presence of 0.5 mM GMP-PNP.

The MS analysis of cross-linking products shown that cpSRP, cpFtsY and ALB3 form a complex with RNC-cytochrome *b*_6_ complexes at the membrane. Furthermore, we have demonstrated that GTP hydrolysis is required for cytochrome *b*_6_ insertion. As presented in Fig. 7B, (lanes 3 and 4) the GMP-PNP (5′ guanylylimidodiphosphate, non-hydrolysable GTP analogue) inhibits cytochrome *b*_6_ integration, suggesting that cpSRP–cpFtsY complex occupies functional ALB3 integration sites.

### ALB3 is crucial for SRP-dependent cytochrome *b*_6_ import into the thylakoid membrane

The SRP-dependent pathway delivers RNC complexes to the Sec/ALB3 translocon^32^,^33^,^34^ or via independent ALB3 insertase in the cytoplasmic membrane^33^. Hence, the results of MS detection of ALB3 suggested that cytochrome *b*_6_ import into the thylakoid membrane was by the Sec/ALB3 insertion site or by ALB3 itself. To test this hypothesis, cell-free native spinach cytochrome *b*_6_ translations were carried out in the presence of thylakoid membrane with the stroma fraction in a presence of ALB3 antibody. We did not observe this effect in the presence of antibody against cytochrome *f* (negative control) or against C-terminus of cytochrome *b*_6_ (Supplemental Fig. S8). The anti-ALB3 serum was able to inhibit the association of a cpSRP–cpFtsY complex with ALB3^28^,^35^,^36^. As shown in Fig. 7A, the translation product import into the thylakoid membrane was significantly reduced if ALB3 function was compromised (Fig. 7A, lane 3). In order to verify the specificity of the anti-ALB3 and to show that it does not block protein insertion into membrane by itself, we tested for PsbW protein spontaneous insertion into membrane in the presence of the anti-ALB3 (Supplemental Fig. S9). This study suggests that ALB3 is crucial for the cytochrome *b*_6_ import into the thylakoid membrane *in vivo.*

## Discussion

The mechanisms responsible for targeting and insertion into the thylakoid membrane of both nuclear and chloroplast-encoded proteins are still not well understood. The presence of signalling sequence, protein structure and origin, are crucial for the choice of transport pathway involved in import process^10^. However, in case of some crucial thylakoid membrane proteins like cytochrome *b*_6,_ the situation is less clear. The chloroplast-encoded cytochrome *b*_6_ is multi-spanning protein without a defined thylakoid membrane signal sequence^37^. Interestingly, it was reported that this protein is able to spontaneously integrate into the bacterial membrane^16^. Furthermore, cytochrome *b*_6_ fusion with an exogenic signalling sequence directed this protein import into a bacterial membrane via the Sec mechanism^18^. The main limitation of these experimental models was the substitution of thylakoid membrane with bacterial as well as the lack of stromal proteins that may facilitate the import process. Furthermore, in the case of the co-translational mechanism of membrane import, the presence of chloroplast-specific proteins may be crucial for cytochrome *b*_6_ folding and orientation within the thylakoid membrane^19^. Hence, to properly examine this protein import into the thylakoid membrane, the model should resemble the chloroplast environment *in vivo*. To address this problem and evaluate the mechanism governing cytochrome *b*_6_ integration *in vivo*, we performed a comparative analysis of both native and synthetic cytochrome *b*_6_ insertion into purified thylakoids. Our criteria for determining the correct mechanism of insertion was (i) that the protein should be integrated with the membrane and hence resistant to chaotropic extraction, (ii) and that it should be properly oriented within the membrane.

As a control for our experimental model, we tested if subunit W (PsbW) of the photosystem II is spontaneously inserted properly into the thylakoid membrane. PsbW is a single-span thylakoid membrane protein that is synthesized with a cleavable hydrophobic signal peptide and integrated into the thylakoid membrane by an apparently spontaneous mechanism^29^. The relatively simple insertion mechanism used by the PsbW together with the notable insertion-competence of the *in vitro* translation products suggested that this protein may be inherently stable in aqueous phases and hence a good model system for studying membrane proteins in general^29^. CD spectroscopy in the ultraviolet wavelength region can be used to estimate this protein secondary structural content and thus the protein folding state. An essential part of the control experiment (insertion of PsbW into the isolated thylakoid membrane) was to show that the factors and methods are actually responsible for the effects observed. This study confirmed previous findings^38^ that for the PsbW proper insertion into the thylakoid membrane, the presence of additional membrane proteins is not required. Furthermore, we also confirmed that PsbW is indeed inserted into the thylakoid membrane by the spontaneous pathway and does not interfere with the ALB3 protein during insertion.

Recently, it was reported that cytochrome *b*_6_ can spontaneously insert into the bacterial cytoplasmic membrane^16^. White and Wimley^39^ proposed a model in which the protein could follow one of two basic spontaneous insertion pathways: a “water path” in which the protein folds (forms α-helices) in the aqueous phase prior to insertion as a folded entity, or an “interface path” where the unfolded protein binds to the membrane interface where α-helix formation is promoted and insertion ensues^29^. Studies of the “water path” are precluded in most cases since the tendency membrane proteins is to aggregate in aqueous buffers. Although, recent studies have shown that the cytochrome *b*_6_ protein is largely unstructured in aqueous solution, it acquires a highly α-helical structure upon interaction with certain types of lipid prior to full insertion^40^.

In our study, the folding of cytochrome *b*_6_ was induced by dialysis of solubilised cytochrome *b*_6_ to buffer with DDM. The disordered structure of the solubilised form of cytochrome *b*_6_ in the presence of DDM was converted into a folded structure^20^.

The formation of α-helices is a key event during spontaneous protein insertion into the membrane. Energetic considerations strongly suggest that these must form prior to full protein insertion^39^,^41^. In contrast, insertion of partially unfolded cytochrome *b*_6_ isolated from *Synechocystis sp.* PCC 6803 was observed nevertheless. Furthermore, completely denatured cytochrome *b*_6_ was only partially imported to the thylakoids and could also be easily removed by urea washes. The formation of α-helices is a key event during membrane protein insertion, but the precise timing of α-helix formation is unclear in most cases^39^. An *in vitro* insertion is extremely difficult to address usually because of the insoluble nature of membrane proteins in aqueous buffer. Herein we show that DDM stabilizes native cytochrome *b*_6_. While soluble cytochrome *b*_6_ showed no detectable α-helix content, upon addition of DDM, there was a rapid increase in secondary structure formation. This indicates that the hydrophobic regions of cytochrome *b*_6_ are able to form α-helical structures in a hydrophobic environment, leading to protein insertion into the membrane^29^. This observation provides strong evidence that apocytochrome *b*_6_ is not able to insert into the thylakoids by a spontaneous pathway.

The Sec-dependent pathway for the integration of cytochrome *b*_6_ into the thylakoid membrane has been also suggested^17^,^24^. However, a lack of the N-terminal presequence in cytochrome *b*_6_ and the opposite orientation in the cytoplasmic membrane after expression in *E. coli* suggests that other pathways are utilized. Indeed, previous studies in bacteria show that the fusion of cytochrome *b*_6_ to MBP (maltose binding protein) directs the cytochrome *b*_6_ onto the Sec-dependent pathway^17^. Hence, topogenic signals in the amino acid sequence of cytochrome *b*_6_ protein are recognised by the *E. coli* Sec translocon leading to integration of this protein into the bacterial inner membrane although in an opposite orientation as compared to that in the thylakoid^18^.

Cytochrome *b*_6_ is not a typical passenger for unassisted integration since it lacks the cleavable N-terminal hydrophobic domain, which is missing in the prokaryotic or plastid-encoded counterparts. Consequently, a “helical hairpin”-type loop insertion mechanism is not feasible for cytochrome *b*_6_. Two variants of an SRP-dependent pathway exist, the posttranslational and the co-translational variant^42^. The SRP-dependent pathway delivers RNC complexes to the Sec translocon or Sec/ALB3 insertion site or to an independent ALB3 in the cytoplasmic membrane. The translocon is an essential component in cotranslational translocation because it binds the RNC and allows the translocation of the nascent chain and coordinates the insertion of proteins into the membrane.

In order to confirm directly that the cytochrome *b*_6_ is a chloroplast protein targeted in a GTP-dependent process termed co-translational translocation, we performed *in vitro* chloroplast import experiments. Although the cytochrome *b*_6_ protein was not synthesized in the wheat germ extracts but in the human *in vitro* protein system, the protein was efficiently transported into the thylakoid membranes. Mass spectrometry analysis of the cytochrome *b*_6_ crosslinked proteins allowed identification of cpSRP54, cpFtsY (both GTPase), ALB3 and CSP41 as potentially involved in targeting and insertion of cytochrome *b*_6_ protein into the thylakoid membrane. As reported previously, cytochrome *b*_6_ elongating nascent chain interacts with the cpSRP54 but not with cpSecY^19^. Hence, the result of crosslinking experiments suggests that in thylakoid, the RNCs contact the SRP in the stroma and then contact the ALB3 protein. Hence, cytochrome *b*_6_ insertion could be co-translational and mediated by cpFtsY and TMH (trans membrane helices) in order to enter the ALB3. cpFtsY would interact with the thylakoid membrane to support cpSRP-dependent targeting. Furthermore, a conserved amphipathic helix located at the N-terminus of cpFtsY that is both necessary and sufficient for interaction with the thylakoid membrane^43^.

We also show that impairment of ALB3 limits cytochrome *b*_6_ import into the thylakoid membrane. These results are consistent with earlier studies showing that nuclease pretreatment did not remove the cytochrome b_6_*-*RNC complexes from the membrane^44^,^45^. Moore, *et al.*^35^ indicated that cpSRP54/cpFtsY copurifies with ALB3 and proposed that cpFtsY and ALB3 form a complex together to insert thylakoid membrane proteins independently of cpSecY. Hence, the ALB3 protein appears to act in two functionally separate pools: one associated with cpSecYEG may serve in cotranslational integration activities, and second functionally independent of cpSecYEG, it mediates integration posttranslationaly^35^. In the chloroplast, ALB3 can function as an insertase independent of other components (cpSecY). ALB3 is exposed rather to the stroma and therefore displays a higher accessibility for stromal components, also beyond the chloroplast SRP (cpSRP) pathway^46^. Nuclear-encoded light-harvesting chlorophyll proteins (LHCP) integrate into the thylakoid membrane using chloroplast SRP proteins and ALB3, but independently of cpSecY^34^. After import into the chloroplast stroma, the LHCP forms with the chloroplast cpSRP the LHCP-cpSRP54-cpSRP43 transit complex, then being directed to the SRP receptor (cpFtsY) at the thylakoid membrane. Binding both cpSRP54 and cpFtsY is a GTP dependent process^35^. At the membrane, the cpSRP43 protein within the transit complex is recognized by ALB3, by utilizing its C-terminal and membrane-embedded domain^47^. In addition to acting independently, ALB3 may insert proteins co-translationally into the thylakoid membrane using cpSec translocase. Specific ALB3′s substrates, that interact with ALB3 using the split-ubiquitin system, are D1, D2, CP43, PSI-A and the ATPase subunit CF_0_III^48^.

However, so far, it has not been shown that these proteins or any other membrane protein strictly requires both ALB3 and cpSecY for insertion into the thylakoid membrane of plants. Moreover, it was also shown that the D1 nascent peptide may also have the ability to localize to the membrane independently of cpFtsY and cpSecY via ALB3. However, the subsequent integration events may require the use of cpSecY for lateral movement or assembly of the D1 protein from the ALB3 translocon into the lipid bilayer^49^.

Herein, we show that thylakoid membrane treatment with anti-cpSecY antibody was not able to prevent the cytochrome *b*_6_ integration. However, antibodies against cpSecY prevent cpSecA-dependent protein translocation^26^,^27^,^28^. This suggest that cpSecY is likely not a part of the functional complex, and cpSRP54 and cpFtsY form the GTP dependent targeting/translocation complex with ALB3.

**Figure 8:**
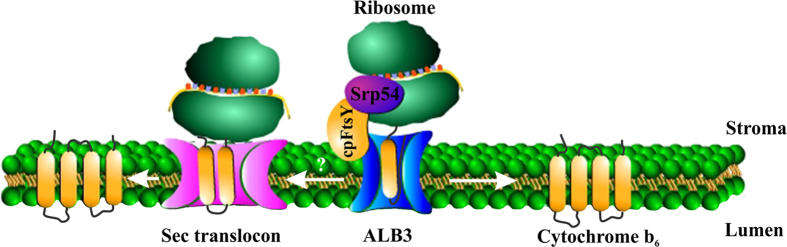
A Model of targeting and insertion of cytochrome *b*_6_ into a thylakoid membrane by using the ALB3 insertase. ALB3 insertase independently of cpSecY imports cytochrome *b*_6_ into the thylakoid membrane. The import process is co-translational and involves cpFtsY and cpSrp54.

Neither this nor previous studies have shown interaction of cytochrome *b*_6_ or RNC-cytochrome *b*_6_ complexes with cpSecY^19^. Hence, we propose that ALB3 insertase independently of cpSecY imports cytochrome *b*_6_ into the thylakoid membrane as depicted in Fig. 8. Although we cannot exclude the involvement of SecYEG translocon, and do not rule out a situation that ALB3 protein may only be involved in an early stage of insertion as assembly factor.

Our mass spectrometry analysis of cytochrome *b*_6_ *in vitro* translation mixtures provided a first evidence that CSP41 interacts with RNC-cytochrome *b*_6_ complexes, and maybe be involved in *petB* transcript stabilization or in a process of monocistronic *petB* mRNA synthesis. CSP41 proteins are highly abundant chloroplast proteins^50^ and ribosome 70S association of CSP41 a and b was reported. The first report on CSP41 suggested that it binds *in vitro* to the 3′ end of the *petD* mRNA^51^. To date multiple functions have been proposed for CSP41proteins: RNase activity, ribosomal biogenesis, and plastid transcriptions^52^. Recently, it was demonstrated by RIP-chip analysis that CSP41 can bind to various chloroplast RNAs and the CSP41 proteins might serve to stabilize RNAs and to protect them against degradation^53^. This includes transcripts for the large Rubisco subunit (*rbcL*), PSI (*psaA, psaB*), and PSII (*psbA, psbC, psbD*) core proteins, and 16S and 23S rRNAs^53^. The lack of CSP41 proteins decreases transcripts for photosynthetic proteins and of some ribosomal RNAs. This included *petD* mRNA, encoding subunit IV from the cytochrome *b*_6_ *f* complex, and is transcribed as a part of the polycistronic cluster *psbB-psbH-petB-petD*, which is subsequently processed to monocistronic mRNAs^54^. In CSP41 compromised mutant *petD* mRNA of the cytochrome *b*_6_ *f* complex was 20% reduced comparing to WT^53^. However, the role of CSP41 in cytochrome *b*_6_ insertion into thylakoid membranes requires further studies.

Herein, we provided evidence for the role of SRP54, FtsY, and ALB3 in targeting and insertion of cytochrome *b*_6_ protein into the thylakoid membrane. Our data demonstrated that the formation of a complex between cpSRP–cpFtsY and ALB3 is a necessary step in the integration of cytochrome *b*_6_ into thylakoid membranes. However, future studies that combine more exact membrane fractionation approaches with RNC profiling are required to understand the interplay between cytochrome *b*_6_ and ALB3 insertase and/or ALB3/SecYEG translocon as well as the role of CSP41.

## Methods

### Isolation Thylakoid Membranes and Stroma from Intact Chloroplasts

Seeds of Pea (*Pisum Sativum*, cv Calvedon) were germinated, planted in plastic trays and grown hydroponically as *described* in ref. 19. Fully developed leaves were harvested 2 h after the lights were turned on. Intact chloroplast from Pea leaves were isolated as described in ref. 19 and total chlorophyll (CHL) content was measured according to^55^.

Washed thylakoids and stromal extract were prepared from isolated chloroplasts as described in ref. 9 except that purified thylakoid membranes (at 1 mg mL^−1^ CHL) were incubated (5 min on ice) with micrococcal nuclease (8 units μL^−1^) in the presence of 10 mM CaCl_2_, protease inhibitor cocktail (P9599, Sigma-Aldrich) and 1 mM DTT (dithiothreitol). The digestion was stopped with 20 mM EGTA. Finally, the isolated thylakoid membranes were washed twice and resuspended in HMS buffer (50 mM HEPES-KOH, pH 8.0 with 10 mM MgCl_2_, 100 mM sorbitol, protease inhibitors cocktail, and 1 mM DTT buffer at 4 mg mL^−1^ of CHL. Reconstituted lysates were prepared fresh prior the experiments by suspending the thylakoid membranes in the stroma.

### Isolation of cytochrome *b*_6_

The isolation of spinach (GenBank: NC_002202.1) apocytochrome *b*_6_ from *E. coli* cells and the protein refolding/reconstitution assays were previously in refs 20 and 24, respectively. Native cytochrome *b*_6_ was isolated from *Synechocystis sp.* PCC 6803 membranes according to^22^. Cytochrome *b*_6_, both native and overexpressed, were biotinylated using the Thermo Scientific EZ-Link PFP-biotin assay.

### Synthetic proteins

The PsbW protein (GenBank: CAA59409.1) was obtained from GenScript at purity above 85%. Cytochrome *b*_6_ as a fusion protein (ss-cytochrome *b*_6_) to the C-proximal part of the signal pre-sequence of the Oxygen Evolving Complex (SLQSDFKELAHKCEASKIAGFALATSALVASGASA, OE33, GenBank: BAA02554.1).

The Ala-X-Ala consensus sequence that is recognised by a thylakoid processing peptidase (TPP) and that cleaves off this transit sequence was changed to protect fusion protein from being cleaved (SLQSDFKELAHKCEASKIAGFALVTSALVASGRSA). An additional Lys was incorporated into C-terminus in order to allow for biotin labelling. The molecular weight of synthetic protein was assessed with the MALDI and ESI-MS techniques (electrospray ionization mass spectroscopy, QTOF Premier mass spectrometer, Waters Corp), using the Mascot database search engine (version 2.1, Matrix Science)^56^.

### Mass spectroscopy

For identification, the proteins were analysed in electrophoresis, stained with Coomassie solution and blotted. Furthermore, protein bands were cut out and subjected to in-gel digestion. After reduction of cysteine residues with DTT and alkylation with iodoacetamide, proteins were digested with trypsin and the resulting peptide mixtures analyzed by liquid chromatography/MS. After preprocessing of the raw data using Mascot Distiller software, output lists of precursor and product ions were compared for identification to the NCBI database using the Mascot database search engine (version 2.1, Matrix Science)^56^. Protein scores are derived from ions scores as a non-probabilistic basis for ranking protein hits. Ions score is −10*Log(P), where P is the probability that the observed match is a random event^56^. We chose only proteins with unique queries. The number of matches MS/MS spectra that uniquely match to the accession and are not shared with other accessions were identified. These matched spectra pass the minimal criteria for ion score and have a false positive rate of less 1%. Furthermore, the analysis resulted in the identification of several hundreds of peptides that were impossible to assign to a specific protein. Therefore, the final analysis score cut off was set at 20 to eliminate low-score peptides, and 40 to eliminate low-score proteins. Individual ions score > 41 indicate identity or extensive homology (p < 0.05)^56^. To calculate total score, the individual ions score with expect to a value lower than 0.05 was chosen for identified peptide (http://www.matrixscience.com/help/scoring_help.html)^56^.

### Protein insertion into thylakoid membrane

In order to remove external endogenous proteins domains, thylakoid membranes were pre-incubated with proteinase K (40 μg mL^−1^ final) for 2 h on ice. The digestion was terminated with 4 mM PMSF (phenylmethylsulfonyl fluoride) and the thylakoid membranes collected (10 min, 11,000 × g). The proteins (0.3 mM final concentration) were incubated with the thylakoid membranes for approximately 1 h. Import incubation mixtures contained the excess of thylakoid membrane (50 μg CHL), stromal extract (30 μg protein) and 10 mM ATP (adenosine 5′-triphosphates). All experiments were carried out at 25 °C under a green safe light, and a microstirrer/heater thermocouple with the reaction medium was used to ensure that the proteins were in a homogeneous distribution and there was a proper temperature within the subphase below the thylakoid membrane. If present, the stromal extract was equivalent to 1.3 times the CHL concentration. The import assays were terminated and unincorporated proteins removed by washing the thylakoid membrane three times in 2 mL of ice-cold 50 mM HEPES-KOH buffer, pH 8.0 with 330 mM sorbitol (3 min, 5,000 × g).

### Proteolytic assessment of protein orientation in thylakoid membranes

The Michl, *et al.*^57^ procedure was used with the following modifications: thylakoid membranes after protein import assays were resuspended in HM buffer (20 mM HEPES-KOH, pH 8.5 mM MgCl_2_, 10 mM KCl and 10 mM DTT) and exposed to light (~300 μmol photons m^−2^ s^−1^) at 4 °C, in order to restore the ΔpH. Subsequently the thylakoid membranes were incubated with trypsin (60 μg mL^−1^, 1,000–2,000 BAEE (Na-benzoyl-arginine ethyl ester) units mg^−1^) for 10 min on ice. The digestion was stopped with trypsin inhibitor (120 μg mL^−1^, Sigma-Aldrich), and membranes were washed twice in HM enriched with 60 μg mL^−1^ trypsin inhibitor (20,000 × *g* for 5 min at 4 °C). The thylakoids were finally collected by centrifugation at 20,000 × *g* for 10 min at 4 °C and resuspended in stromal extract or in 15 μL of HM buffer containing 5 μg of trypsin inhibitor.

Thylakoid membranes prior to carboxypeptidase B (C9584, Sigma-Aldrich) treatment were supplemented with procaine and Triton X-100 at final concentrations of 3 mM and 0.01%, respectfully. Then, carboxypeptidase B (5 mg/mL, ≥125 units mg^−1^ protein) in 0.1 M Na^+^ citrate buffer, pH 6.5) was added for 1 h at 30 °C. Subsequently, after this treatment an equal aliquot of enzyme solution was added and the incubation continued for another 30 min. Controls received 0.1 M Na^+^ citrate buffer, pH 6.5, instead of the enzyme solution. The final pH of the samples was kept at 6.5–7.0. The proteolytic reaction was stopped by addition of the carboxypeptidase inhibitor (C0279, Sigma-Aldrich).

### Assessment of integration of the targeted proteins into the thylakoid membrane

The proteins associated with but not integrated into the thylakoid membranes are not resistant to chaotropic agents^23^. To monitor integration of the targeted proteins, the thylakoid membranes (at 0.1 mg mL^−1^ CHL) were incubated for 15 to 30 min on ice in either 0.1 M NaHCO_3_-NaOH, pH 12.5 or 4 M urea in 50 mM HEPES-KOH pH 7.7, 50 mM potassium acetate, 100 mM mannitol, 5 mM magnesium acetate, 2 mM DTT and a protease inhibitor cocktail. After these incubations, the membranes were washed and collected in HMS buffer (16,000 × g for 3 min).

### Cell free transcription-translation assay

Coupled transcription/translation reactions of spinach cytochrome *b*_6_ construct were performed with the Human *in vitro* Protein Expression Kit for DNA Templates (Thermo Scientific). For *in vitro* transcription of the full length cytochrome *b*_6_ plasmid pT7CFE1-*b*_6_ containing the 5′ UTR consisting of EMCV internal ribosome entry site (IRES) was used. pT7CFE1-*b*_6_ was isolated using a standard mini prep protocol (GeneJET Plasmid Miniprep Kit, K0502, ThermoFisher Scientific). To avoid compromising protein expression yield, RNase A was used during the purification. Transcription with T7 polymerase was performed according to Human *in vitro* Protein Expression Kit for DNA. The reaction was performed at 32 °C for 90 min in a 20 μL reaction mixture and a total of 1 μg of DNA template was used. The mRNA (2 μg) generated from the transcription was added to each translation reaction mix containing all of the machinery for protein expression (cell lysate for protein expression, accessory proteins, amino acid minus methionine, energy mix). Translation reactions were performed essentially according to manufacturer’s recommendations for a 50 μL translation reaction. However, some modifications were made for insertion of cytochrome *b*_6_ into thylakoid membrane. Radioactive [^35^S]methionine (1,000 Ci/mmol) and additional RNase and protease inhibitors (RNasin, antipain, pepstatin and leupeptin, 0.2 mM each) were used during the translation assays. Mannitol was added (100 mM) to stabilize thylakoid membrane. Cytochrome *b*_6_ was synthesized *in vitro* in the presence of thylakoid membrane (250 μg mL^−1^ of CHL) with or without the fresh prepared stromal fraction (equivalent to thylakoid membrane containing 250 μg mL^−1^ of CHL) for 2 h at 30 °C. To increase protein yield, 2 μL more of the transcription mixture (1.0 μg μL^−1^) and 1 μL of energy mix was added for each reaction being performed after 2 h of translation and each reaction mixture was incubated for another 2 hours. The total reaction volume was 100 μL. Each translational sample (25 μL) was analysed by SDS-PAGE and the autoradiography.

In order to remove external domains of endogenous proteins, thylakoid membranes or stroma were pre-incubated with proteinase K (40 μg mL^−1^ final) for 2 h on ice. The digestion was terminated with 4 mM PMSF and the thylakoid membranes collected (10 min, 11,000 × g). In order to remove endogenous RNA during translation reactions thylakoid membranes or stroma were pre-incubated with RNase A (5 ng RNase A) for 15 min at 37 °C. Prior to translation reaction ribonuclease inhibitor (RNasin, Promega) was added (2 μl RNasin ribonuclease inhibitor (40 units/μL).

### Co-immunoprecipitation

To isolate targeted RNCs, translation reactions were diluted with one volume of ice-cold 50 mM HEPES-KOH, pH 7.8, 1 M potassium acetate, 10 mM magnesium acetate buffer (HMS) and centrifuged for 1 min at 12,000 × g. The thylakoid membranes pellets were re-suspended to the original volume (100 μl) in HMS buffer and treated (for 30 min at 0 °C) with the membrane-permeable crosslinkers: BSOCOES (homobifunctional *N*-hydroxysuccinade ester bis[2-(succinimidyloxycarbonyloxy) ethyl] sulfone, Pierce) or DMA (homobifunctional imidoester dimethyladipimidate, Pierce). Reactions were then quenched with a large molar excess of DTT in an equal volume of HMS buffer. The thylakoid membranes were than incubated with 1% DDM (30 min at 0 °C) and insoluble materials were removed by ultracentrifugation at 50,000 rpm (Beckman SW60 Ti rotor) at 4 °C for 1 h. The resultant supernatant was further incubated (30 min) with an excess (5 μg mL^−1^) of antibody against cytochrome *b*_6_ N-terminus^19^. Subsequently, protein A- Sepharose CL-4B (GE Healthcare) was added and incubation was continued for an additional 4 h with rotation at 4 °C. The resin was collected (500 × g, 5 min, 4 °C) and washed three times with two volumes of HMS buffer. After the final wash, the bound protein fraction was eluted (in 0.25 M Tris-HCl, pH 6.8 with 100 μL 5% SDS, 8 M urea, for 1 h at 37 °C), analysed in SDS-PAGE, and subjected to autoradiography^58^. Furthermore, the detected bands were excised from a polyacrylamide gel and analysed by mass spectroscopy with fingerprints.

### Spectra measurements and circular dichroism analyses

PsbW and cytochrome *b*_6_ were solubilised in 1% DDM. To remove the free detergent, samples were extensively dialyzed against HM buffer and then centrifuged at 100,000 × g to remove any insoluble aggregates. Far UV and VIS CD spectra were performed as described in ref. 24. To gain information on the secondary structure, the CD spectra were analysed^59^.

### Autoradiography and Western blot

The protein concentration was determined with the BCA (bicinchoninic acid) reagent according to the manufacturer’s instructions (Pierce). Following the normalization of protein concentrations, samples were mixed with an equal volume of 2X Laemmli sample buffer and incubated for 5 min at 95 °C prior to separation by Tricine/Tris SDS-PAGE as described in ref. 60. Prior to autoradiography, gels were stained with Coomassie brilliant blue, to confirm equal protein loading and dried under vacuum. The X-ray film was placed over the dry gel and exposed for 24 h. Importantly, we always used the same exposure time so that the image intensity was used as a consistent guide to the exposure required for subsequent autoradiography. Following SDS-PAGE, the proteins were transferred to nitrocellulose membranes (180 mA for 30 min at 4 °C). The membranes were then blocked with bovine serum albumin (BSA, Sigma-Aldrich) in PBS/Tween-20 (3% BSA, 0.5% Tween-20) for 1–2 hours, followed by immunoblotting with the primary antibody specified for each experiment: antibodies against N-terminal residues of cytochrome *b*_6_ as well as against the interhelical part of cytochrome *b*_6_ (between helix 1 and 2 and between helix 3 and 4)^19^, and against ALB3^36^ were prepared in rabbits injected with the synthetic peptide (GenScript); biotin antibody was purchased from Abcam^®^ (ab53468). After washing steps (3 washes with 20 mM sodium bicarbonate buffer pH 7.4, 5 min each at room temperature, followed by 3 washes with 100 mM glycine pH 2.4, 10 min each, at room temperature) the membranes were incubated with goat anti-rabbit IgG (H + L) secondary antibodies (BioRad) and detected using ECL (enhanced chemiluminescence, Amresco). Densitometry was performed using Image Lab software v. 4.1 (BioRad). We use the same exposure time for all autoradiography.

## Additional Information

**How to cite this article**: Króliczewski, J. *et al.* ALB3 Insertase Mediates Cytochrome *b*_6_ Co-translational Import into the Thylakoid Membrane. *Sci. Rep.* **6**, 34557; doi: 10.1038/srep34557 (2016).

## Supplementary Material

Supplementary Information

## Figures and Tables

**Table 1:** Predicted UV-CD secondary structure analysis of native and unfolded cytochrome *b*_6_ isolated from *Synechocystis sp.* PCC 6803.

Structure	Native cytochrome *b*_6_	Unfolded cytochrome *b*_6_
α-helix	50.29 ± 1.4	19.22 ± 0.8
β-sheet	24.10 ± 0.7	18.23 ± 1.3
β-turn	8.17 ± 0.3	5.51 ± 0.6
Random coil	17.44 ± 0.9	57.13 ± 1.4

Calculated values are the average of four independent measurements, presented as means ± standard deviation.

**Table 2:** The selected proteins crosslinked to cytochrome *b*_6_ RNC complexes identified by mass spectroscopy and peptide mass fingerprinting.

No.	Protein^a^	Total Protein score^c^	Peptide identified by MS^b^	Annotation in database	Function
1	cpFtsY	335	VLDELEEALLVSDFGPKITVR LREDIMSGK ESVLEMLAK EFNEVVGITGLILTK	Arabidopsis thaliana, CAB40382.1	SRP-membrane-associated receptor of translating ribosomes^43^.
2	cpSRP54	524	FDFNDLLK ILGMGDVLSFVEK TEQQVSQLVAQLFQMR QVDVPVYAAGTDVKPSVIAK NLQFMEVIIEAMTPEER FLNPTEVLLVVDAMTGQEAA-ALVTTFNVEIGITGAILTK	Pisum sativum AAC64109.1	cpSRP54 has both a role in posttranslational targeting of nuclear-encoded thylakoid proteins, and has also been implicated in co- translational targeting and insertion into the thylakoid membrane.
3	ALB3	419	ALQQRYAGNQER SLAQPDDAGER AATYPLTK YAGNQER	Arabidopsis thaliana AEC08172.1	Required for the insertion of integral membrane proteins into the thylakoid inner membrane^61^. ALB3 plays a role in the co-translational integration of the D1 protein into the thylakoid membrane, although the exact mechanism of this localization and integration is unknown^49^
4	CSP41	319	QFLFISSAGIYK SSGVKQFLFISSAGIYK DCEEWFFDRIVR DRPVLIPGSGMQLTNISHVKD	Arabidopsis thaliana Q9LYA9.1	Bind and stabilize distinct plastid transcripts, complexes. CSP41 proteins stabilize untranslated mRNAs and precursor rRNAs^52^.

All experiments were repeated at least two times. The complete list of identified proteins and detailed detection parameters are provided in Supplemental Table S1.

^a^Chloroplast proteins which produced the highest scores are shown.

^b^The study also showed several hundred peptides impossible to assign to a specific protein, therefore we set a score cut off at 20 to eliminate low-score peptides, and at 40 to eliminate low-score proteins.

^c^Individual ions score > 41 indicate identity or extensive homology (p < 0.05). To calculate total score, only the individual ions score with expect value less than 0.05 was chosen for the identified protein.
