# Supplementary material for: ALB3 Insertase Mediates Cytochrome b6 Co-translational Import into the Thylakoid Membrane
Source: Sci Rep. 2016 Oct 4;6:34557. doi: 10.1038/srep34557 (PMC5048292; doi:10.1038/srep34557)
Supplement: Supplementary Information [file srep34557-s1.pdf]

Supplementary Information

ALB3 insertase mediates cytochrome b_6 co-translational import into the thylakoid membrane

Jarosław Króliczewski[1], Małgorzata Piskozub[2,3], Rafał Bartoszewski[3], Bożena Króliczewska[4].

[1]Laboratory of Chemical Biology, Faculty of Biotechnology, University of Wrocław, Wrocław Poland.

[2]Amplicon Sp. z o. o., Wrocław, Poland.

[3]Faculty of Biotechnology, University of Wrocław, Wrocław, Poland.

[4]Department of Biology and Pharmaceutical Botany, Medical University of Gdansk, Gdansk, Poland

[5]Department of Animal Physiology and Biostructure, Faculty of Veterinary Medicine, Wroclaw University of Environmental and Life Sciences, Wrocław, Poland

Correspondence should be addressed to J.K (email: jakrol@windowslive.com)

```
                    1                                                50
Pisum sativum   (1) -------MSKVYDWFEERLEIQAIADDITSKYVPPHVNIFYCLGGITLTC
Syn 6803        (1) MFSKEVTESKVFQWFNDRLEVQAISDDIASKYVPPHVNIFYCLGGLTLTC

                    51                                              100
Pisum sativum  (44) FLVQVATGFAMTFYYRPTVTEAFASVQYIMTEANFGWLIRSVHRWSASMM
Syn 6803       (51) FLIQFATGFAMTFYYKPTVTEAFASVQYIMNEVNFGWLIRSIHRWSASMM
                    101                                             150
Pisum sativum  (94) VLMMILHVFRVYLTGGFKKPRELTWVTGVVLGVLTASFGVTGYSLPWDQI
Syn 6803      (101) VLMMILHVFRVYLTGGFKKPRELTWVVGVMLAVTTVTFGVTGYSLPWDQV
                    151                                             200
Pisum sativum(144)  GYWAVKIVTGVPDAIPVIGSSVVELLRGSASVGQSTLTRFYSLHTFVLPL
Syn 6803      (151) GYWAVKIVSGVPAAIPVVGDQLVTLMRGSESVGQATLTRFYSLHTFVLPW
                    201                 222
Pisum sativum(194)  LTAVFMLMHFPMIRKQGISGPL
Syn 6803      (201) AIAVLLLLHFLMIRKQGISGPL
```

Figure S1. Sequence alignment of **PetB** ***from Pea (Pisum sativum) and Synechocystis sp. PCC 6803.*** Fully conserved residues are shaded in yellow, similar residues are shaded in green.

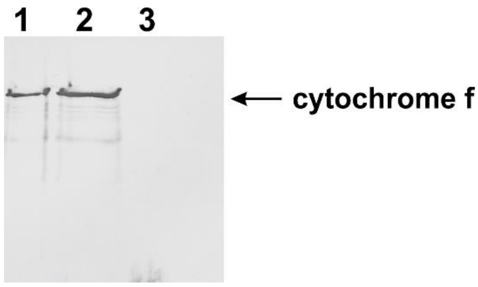

Figure S2. Western blot analysis of extraction of thylakoid membrane proteins with inserted cytochrome b_6 by urea. Pellet and supernatant were analyzed by SDS-PAGE followed by immunoblotting using antisera against cytochrome *f*. Lane 1, thylakoid membranes after insertion of cytochrome b_6; lane 2, pelleted thylakoid membranes after chaotropic treatment; lane 3, supernatant fraction of pelleted thylakoid membranes.

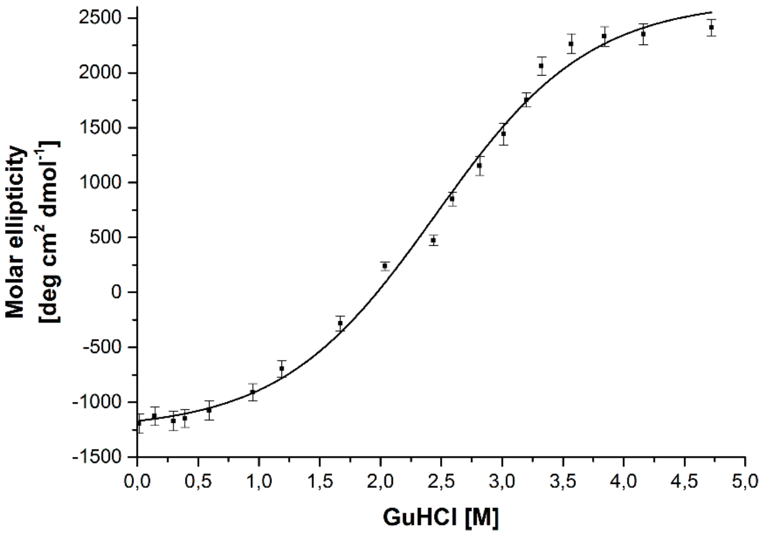

Figure S3. Refolding of E. coli overexpressed spinach apocytochrome b_6. The normalized ellipticity at 222 nm is plotted against GuHCl concentration and a sigmoidal curve fitted. Error bars are standard deviations (SD) from n = 3 replicate analyzes. Statistical analysis was performed with Statistica 10 software. Results were expressed as means ± SD.

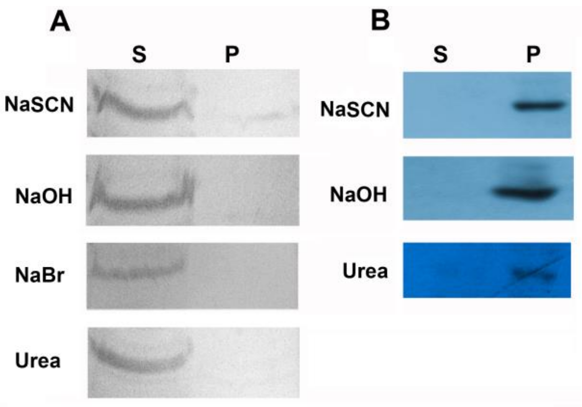

Figure S4. Analysis of extraction of inserted cytochrome b_6 into thylakoid membraneh. **(A)** Extraction of cytochrome *b*$_6$ inserted by spontaneous pathway by solutions of chaotropic salts or alkaline pH. Pellet and supernatant were analyzed by SDS-PAGE followed by immunoblotting using biotin antisera. **(B)** Extraction of cytochrome *b*$_6$ by solutions of chaotropic salts or alkaline pH. Chloroplast import experiments were performed using a cell free *in vitro* system. Pellets and supernatants were analyzed by SDS-PAGE followed by immunoblotting using antisera against the N-terminus of cytochrome *b*$_6$.

After incubation for 30 min on ice, the assays were separated into membrane fraction (P) and supernatants (S).

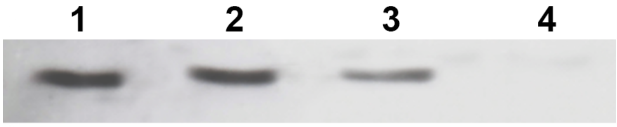

Figure S5. In vitro **import of ss-cytochrome *b_6* into thylakoid membrane.** The integration of the biotin labelled ss-cytochrome b_6 into the thylakoid membrane in the presence of stromal fraction and antibody against cpSecY was analysed with Western blot. Lane 1, ss-apocytochrome b_6 as a control; lane 2, ss-apocytochrome after insertion into thylakoid membrane in the presence of stromal fraction; lane 3 supernatant and lane 4 membrane pellet after fractionation of ss-apocytochrome b_6 inserted into membranes in the presence of stroma and cpSecY antibody. The fusion signal sequence of ss-apocytochrome b_6 lack sequence that is recognised by a thylakoid TPP (thylakoid processing peptidase). An antibody against biotin was used for detection.

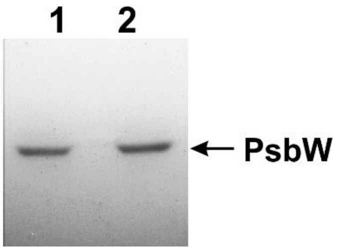

Figure S6. Insertion of PsbW into isolated thylakoids in the presence of apyrase. Western blot analysis of PsbW protein after insertion into thylakoid membranes. Membranes and stroma was treated with apyrase before PsbW insertion. The solution was centrifuged at 27,000 x g for 5 min to pellet the membranes. The pelleted thylakoid membranes were washed twice and analysed by SDS-PAGE and Western blot. Lane 1, control sample containing PsbW protein inserted into thylakoid membranes in the presence of untreated stromal fraction; lane 2, thylakoid membranes after insertion of PsbW into thylakoid membranes. Antibody against biotin was used.

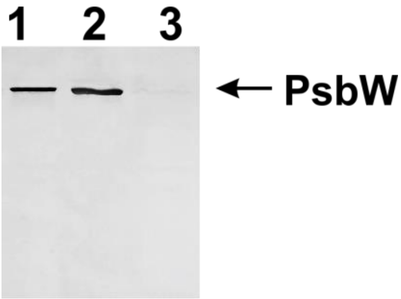

Figure S7. Thylakoid membrane fractions after insertion of PsbW and treatment with chaotropic agents. Lane 1. thylakoid membranes after insertion; lane 2. pelleted by centrifugation thylakoid membranes after chaotropic treatment; lane 3, supernatant after centrifugation of chaotropic treatment thylakoid membranes; antibody against biotin was used. Densitometric analysis showed that more than 99% of PsbW protein is not extractable by chaotropic treatment.

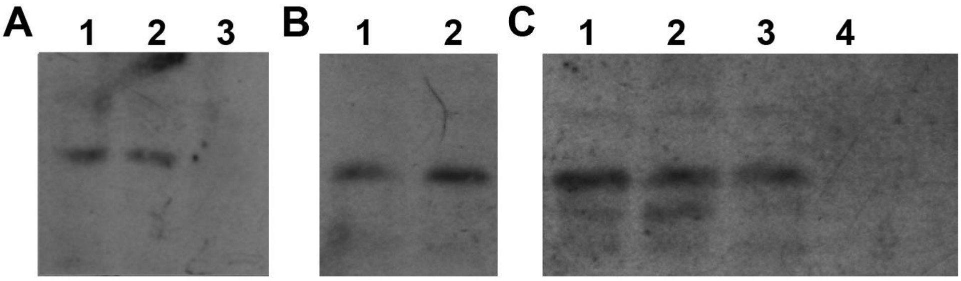

Figure S8. Autoradiograph of cytochrome b_6 expressed in cell free assay in the presence of thylakoid membrane and stroma. **(A)** Lane 1, translation of cytochrome *b*$_6$ - control; lane 2 and 3, translation of cytochrome *b*$_6$ in the presence of thylakoid membrane, stromal fraction and cpSecY antibody, membrane pellet and supernatant after fractionation, respectively; **(B)** Lane 1, membrane pellet after translation of cytochrome *b*$_6$ in the presence of thylakoids membrane, stroma and antibody against cytochrome *f* (negative control); lane 2, translation of cytochrome *b*$_6$ in the presence of thylakoid membrane and stromal fraction; **(C)** Lane 1, translation of cytochrome *b*$_6$ - control; lane 2, translation of cytochrome *b*$_6$ in the presence of thylakoid membrane, stromal fraction and C-terminus cytochrome *b*$_6$ antibody, membrane pellet after fractionation; lane 3 and 4, translation of cytochrome *b*$_6$ in the presence of thylakoids membrane, stromal fraction, ALB3 antibody supernatant and membrane pellet, respectively after fractionation.

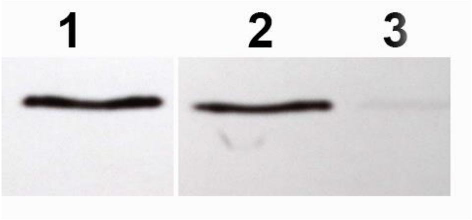

Figure S9. Insertion of PsbW into isolated thylakoids in the presence of anti-ALB3. Indicated proteins were detected by Western blot analysis using an anti-biotin antibody. Lane 1, control sample containing PsbW protein before insertion; lane 2, thylakoid membranes after insertion of PsbW into thylakoid membranes; lane 3, supernatant after fractionation by centrifugation.

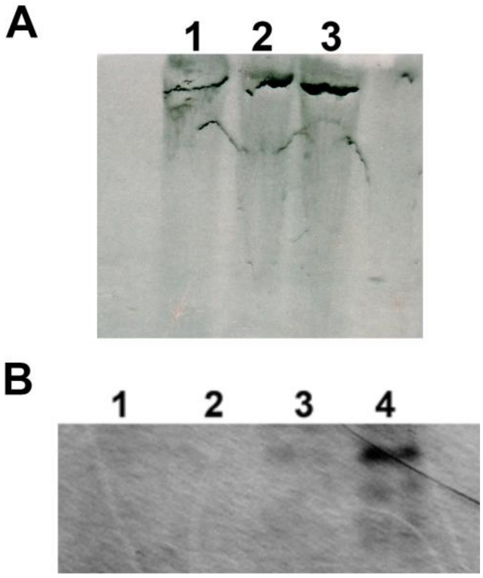

Figure S10. Autoradiograph of cytochrome b₆ expressed in cell free assay. **(A)** Cytochrome b_6 expressed in cell free assay without the presence of thylakoid membrane and stroma fraction Expression samples from different time points. Lane 1, 1,5 h; lane 2, 2 h; lane 3, 3 h. **(B)** Autoradiograph of cytochrome b_6 expressed in cell free assay in the presence of thylakoid membrane and stroma fraction. Expression samples from different time points. Lane 1, 30 min; lane 2, 1 h; lane 3, 1.5 h, lane 4, 3 h.

Table S1. ***Identification of proteins by ESI-MS/MS from crosslinked RNC-cytochrome b_6 complexes.***

Accesion ver.	Protein	Peptides	Score	Expect value	[a]Total Score	[b]Queries matched
P08241.1	30S ribosomal protein S2, chloroplastic	QFLIVGTK<br>GMLTNWYTTETR<br>QLSHFETYLGGIK<br>YMTGLPDIVHVDQQK	46<br>92<br>70<br>114	0.02<br>3.9e-07<br>0.00013<br>4.3e-09	322	10
P31165.1	50S ribosomal protein L15, chloroplastic;	SGPGIMR<br>AGAFSTSAK<br>AEEYFAK<br>GFEGGQMPLYR<br>GIAAGQGASCGFGMR<br>FRLDNLGPQPGSR	53<br>57<br>41<br>87<br>93<br>55	0.008<br>0.0018<br>0.032<br>2.2e-06<br>3.1e-07<br>00035	386	14
NP_192040.1	50S ribosomal protein L5 family protein	LLALMGMPFR<br>SDQEGQKLLALMGMPFR	63<br>73	0.00063<br>5.8e-05	136	11
AAO84599.1	30S small ribosomal protein S4	GSTGEVLLQLLEMR<br>LGMAATIPQAR<br>TLIQNSLESAPR	65<br>45<br>97	0.0004<br>0.04<br>2.6e-07	207	10
AAG23857.1	50S ribosomal protein L2	IITIEYDPNR<br>GAIIGDTIVYGTEVPIK<br>MGNALPLTDMPLGTAIHNIEITLGK	48<br>111<br>47	0.024<br>6.1e-09<br>0.015	206	7
P17092.1	30S ribosomal protein S17, chloroplastic	VGDIVQLLK<br>AGSSGELGIPLQSQQE<br>KAGSSGELGIPLQSQQE	84<br>52<br>45	2.3e-06<br>0.0078<br>0.035	181	10
P11893.1	50S ribosomal protein L24, chloroplastic	HNSSVTVK<br>SNQEGEPGQINK	64<br>52	0.00024<br>0.0057	116	2
YP_001381693.1	50S ribosomal protein L14, chloroplastic	YDDNAAVLIDK<br>MIQPQTYLNVADNSGAR	74<br>89	4.9e-05<br>1.5e-06	163	3
O80360.1	50S ribosomal protein L3, chloroplastic;	QLGSIGAGTTPGR.<br>EGDLVDVSGTTIGK<br>TEATDGYNAVQVGYR<br>EGDLVDVSGTTIGKGFQGGIK	64<br>48<br>134<br>48	0.00052<br>0.023<br>3.3e-11<br>0.023	294	13
YP_002149767.1	30S ribosomal protein S8	DTIADILTLIR<br>VLGGMGIVTLSTSR<br>TVQIPLTNITENIVK	68<br>59<br>78	0.00012<br>0.00093<br>5.6e-06	205	4
P00155.2	Cytochrome f	NILVIGPVPGK<br>YSEITFPILSPDPATK<br>GGYEITIVDASDGR	48<br>77<br>54	0.003<br>2.1e-05<br>0.0048	179	7
P49208.1	50S ribosomal protein L1, chloroplastic	GGFMEFDK<br>YNDQQLR<br>VAVLTQGER<br>LIASPDMMPK<br>FVETVEAHFR<br>NAGADLVGGEDLIEQIK	54<br>57<br>59<br>60<br>48<br>103	0.00065<br>0.0027<br>0.0021<br>0.0012<br>0.0027<br>5.2e-08	381	15
AEC08172.1	ALBINO3<br><br>(ALB3)	ALQQRYAGNQER<br>SLAQPDDAGER<br>AATYPLTK<br>YAGNQER	95<br>129<br>106<br>89	4.5e-07<br>2.3e-09<br>1.2e-05<br>0.00015	419	14

XP_002280604.1	30S ribosomal protein S1	IAQAEAMAR<br>VMILSHDR<br>AEEMAQTFR<br>FVEVDEEQSR<br>SIQYDLAWER	72<br>52<br>74<br>65<br>82	0.00012<br>0.005<br>2.6e-05<br>0.00029<br>1.1e-05	345	14
AAC64109.1	signal recognition particle 54 kDa subunit precursor	GGAALSVK<br>ILGMGDVLSFVEK<br>TEQQVSQLVAQLFQMR<br>MEDLEPFYPDR<br>FLNPTEVLLVVDAMTGQEAAALVT-<br>TFNVEIGITGAILTK	72<br>100<br>101<br>56<br>109<br>86	4.4e-05<br>1.1e-07<br>9.4e-08<br>0.001<br>8.3e-10<br>5.8e-06	524	28
CAB40382.1	chloroplast<br><br>FtsY homolog	VLDELEEALLVSDFGPKITVR<br>LREDIMSGK<br>ESVLEMLAK<br>EFNEVVGITGLILTK	114<br>63<br>75<br>83	5e-05<br>0.00032<br>2.4e-05<br>2.8e-06	335	13
Q6KGX3.1	30S ribosomal protein S7, chloroplastic	VVNMLVNR<br>LSSELVDAAK<br>VSGSTHQVPIEIESTQGK	51<br>46<br>53	0.012<br>0.041<br>0.0056	150	5
Q9LYA9.1	chloroplast stem–loop-binding protein (CSP41)	QFLFISSAGIYK<br>SSGVKQFLFISSAGIYK<br>DCEEWFFDRIVR<br>DRPVLIPGSGMQLTNISHVKD	79<br>105<br>60<br>75	1.3e-05<br>2.6e-08<br>0.0011<br>3.4e-05	319	12
P13911.1	Plastid-encoded RNA polymerase subunit alpha	VSTQTLQWK<br>GQADTIGITMR<br>SNIHTVLELLNK<br>FILNILQIENHFV<br>TLNNIQDGSYTIDAVFMPVR	61<br>93<br>81<br>99<br>135	0.0011<br>9.7e-05<br>4.8e-07<br>1.1e-07<br>3.2e-11	469	18
NC_002202.1	cytochrome b_6	VYDWFEER<br>SVHRWSASMMVLMMILHVFR<br>VYLTGGFKKPR<br>IVTGVPDAIPVIGSPLVELLR<br>FYSLHTFVLPLLTAVFMLMHFLMIR<br>LEIQAIADDITSK	82<br>114<br>58<br>95<br>124<br>71	6.7e-06<br>2.5e-09<br>0.00018<br>1.4e-05<br>3.2e-09<br>9.6e-05	544	21
P23408.1	50S ribosomal protein L22, chloroplastic	GLPDESDKEENSS	41	0.019	41	3
P06587.1	30S ribosomal protein S11, chloroplastic	GTPFAAQTAAGNAIQTVVEQGMQR	43	0.05	43	2
NC_014057.1	subunit IV	MGVTKKPDLTDPVLR<br>AKLAK	84<br>46	4.2e-05<br>0.0018	130	3

[a]proteins with higher scores were not observed after cross-linking.
[b]Total number of queries: 2780

Protein scores are derived from ions scores as a non-probabilistic basis for ranking protein hits. Ions score is −10*Log(P), where P is the probability that the observed match is a random event. We chose only proteins with unique queries. The number of matches MS/MS spectra that uniquely match to the accession and is not shared with other accessions that were identified. These matched spectra pass the minimal criteria for ion score and have a false positive rate of less 1%.

Furthermore, the analysis resulted in identification of several hundreds of peptides, that were impossible to assign to a specific protein. Therefore, for the final analysis a score cut off was set at 20 to eliminate low-score peptides, and 40 to eliminate low-score proteins. Individual ions score > 41 indicate identity or extensive homology ($p<0.05$). To calculate total score, the individual only ions score with expect value less than 0.05 was chosen for identified protein.

Search Parameters

Type of search: MS/MS Ion Search
Enzyme: Trypsin
Fixed modifications: Carbamidomethyl (C)
Variable modifications: Oxidation (M), Carbamidomethyl (K)
Mass values: Monoisotopic
Protein Mass: Unrestricted
Peptide Mass Tolerance: ± 40 ppm
Fragment Mass Tolerance: ± 0.8 Da
Max Missed Cleavages: 1
Instrument type: ESI-TRAP
Database: NCBInr
Taxonomy: Viridiplantae (Green Plants) (730741 sequences)

Table S2. *Analysis of proteins co-immunoprecipitated with RNC-cytochrome b_6 after in vitro translation.* Immunoprecipitation of RNC-cytochrome b_6 complexes using an antibody against cytochrome b_6 covalently linked to Protein A/G-coated beads. Bound proteins were directly analysed by ESI-MS/MS. An antibody against cytochrome b_6 N-terminus was used. a crosslinker was not used for co-immuoprecipitatation.

Accesion ver.	Proteins[a]	Score	Queries matched[b]	emPAI
NC_002202.1	*Cytochrome b_6 (chloroplast) [Spinacia oleracea]*	825	41	1.07
EEE70519.1	*Predicted protein [Populus trichocarpa]*	122	2	0.09
XP_002280604.1	*Ribosomal protein S3,*	54	3	0.21
XP_001698945.1	*Hypothetical protein [Chlamydomonas reinhardtii]*	56	1	0.06
NP_198930.1	*Unknown protein [Arabidopsis thaliana]*	51	1	0.02
EEH53252.1	*Predicted protein [Micromonas pusilla CCMP1545]*	49	2	0.07
XP_001771116.1	*Predicted protein [Physcomitrella patens subsp. Patens]*	49	1	0.08
XP_002538756.1	*Dihydrolipoamide acetyltransferase component of pyruvate dehydrogenase, putative [Ricinus communis]*	48	1	0.28
AAY68198.1	*RNA polymerase IV second largest subunit [Rhododendron macrophyllum]*	47	3	0.03
CAN64973.1	*Hypothetical protein [Vitis vinifera]*	46	1	0.03
EEF09531.1	*Predicted protein [Populus trichocarpa]*	46	1	0.05
XP_002502311.1	*Kinesin-like protein FLA8 [Micromonas sp. RCC299]*	46	1	0.02
CAN78012.1	*Hypothetical protein [Vitis vinifera]*	45	1	0.04
XP_001417143.1	*Predicted protein [Ostreococcus lucimarinus CCE9901]*	45	1	0.06
NP_189250	*CYP71B21; electron carrier/ heme binding / iron ion binding / monooxygenase/ oxygen binding [Arabidopsis thaliana]*	45	1	0.03
EEH57564.1	*Predicted protein [Micromonas pusilla*	45	1	0.03

	CCMP1545]			
ABK95605.1	*unknown [Populus trichocarpa]*	44	1	0.03
XP_002338502.1	*Predicted protein [Populus trichocarpa]*	40	2	0.05
Q9M571.1	*Phosphoethanolamine N-methyltransferase*	43	1	0.16
XP_001416609.1	*Predicted protein [Ostreococcus lucimarinus CCE9901]*	40	1	0.06
1ULN_A	*Chain A, Crystal Structure of Pokeweed Lectin-D1*	32	1	0.34
XP_001777275.1	*Predicted protein [Physcomitrella patens subsp. patens]*	0	1	0.00

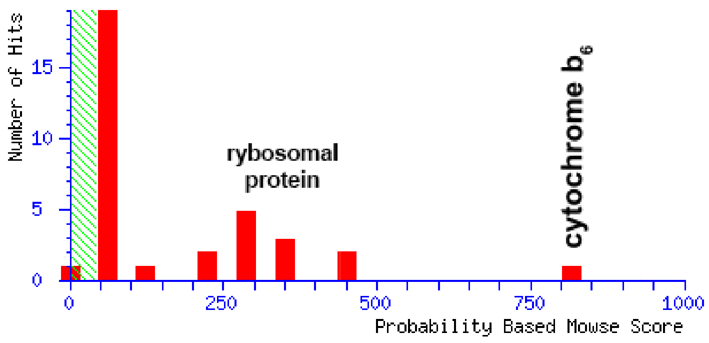

Probability Based Mowse Score
Ions score is -10*Log(P), where P is the probability that the observed match is a random event.
Individual ions scores > 41 indicate identity or extensive homology (p<0.05).
Protein scores are derived from ions scores as a non-probabilistic basis for ranking protein hits.

[a]Furthermore, ribosomal proteins have been identified with total score from 50 to 450
[b]Peptide matches not assigned to protein hits: 570

Search Parameters

Type of search: MS/MS Ion Search
Enzyme: Trypsin
Fixed modifications: Carbamidomethyl (C)
Variable modifications: Oxidation (M), Carbamidomethyl (K)
Mass values: Monoisotopic
Protein Mass: Unrestricted
Peptide Mass Tolerance: ± 40 ppm
Fragment Mass Tolerance: ± 0.8 Da
Max Missed Cleavages: 1
Instrument type: ESI-TRAP
Database: NCBInr
Taxonomy: Viridiplantae (Green Plants) (730741 sequences)
